# Ultralow radiant exposure of a short-pulsed laser to disrupt melanosomes with localized thermal damage through a turbid medium

**DOI:** 10.1038/s41598-024-70807-7

**Published:** 2024-08-29

**Authors:** Yu Shimojo, Takahiro Nishimura, Daisuke Tsuruta, Toshiyuki Ozawa

**Affiliations:** 1 Derpartment of Dermatology, Graduate School of Medicine, Osaka Metropolitan University, 1-4-3 Asahimachi, Abeno, Osaka, 545-8585 Japan; 2 Graduate School of Engineering, Osaka University, 2-1 Yamadaoka, Suita, Osaka 565-0871 Japan; 3 Research Fellow of Japan Society for the Promotion of Science, 5-3-1 Kojimachi, Chiyoda, Tokyo, 102-0083 Japan

**Keywords:** Biomedical engineering, Biophotonics

## Abstract

Short-pulsed lasers can treat dermal pigmented lesions through selective photothermolysis. The irradiated light experiences multiple scattering by the skin and is absorbed by abnormal melanosomes as well as by normal blood vessels above the target. Because the fluence is extremely high, the absorbed light can cause thermal damage to the adjacent tissue components, leading to complications. To minimize radiant exposure and reduce the risk of burns, a model of the melanosome-disruption threshold fluence (MDTF) has been developed that accounts for the light-propagation efficiency in the skin. However, the light-propagation efficiency is attenuated because of multiple scattering, which limits the extent to which the radiant exposure required for treatment can be reduced. Here, this study demonstrates the principle of melanosome disruption with localized thermal damage through a turbid medium by ultralow radiant exposure of a short-pulsed laser. The MDTF model was combined with a wavefront-shaping technique to design an irradiation condition that can increase the light-propagation efficiency to the target. Under this irradiation condition, melanosomes were disrupted at a radiant exposure 25 times lower than the minimal value used in conventional laser treatments. Furthermore, almost no thermal damage to the skin was confirmed through a numerical simulation. These experimental and numerical results show the potential for noninvasive melanosome disruption and may lead to the improvement of the safety of short-pulsed laser treatment.

## Introduction

The use of short-pulsed lasers is effective for treating dermal pigmented lesions^1,2^. The principle of treatment is largely based on selective photothermolysis^3^. The pulse width is selected in the range from picoseconds to nanoseconds, which is shorter than the thermal relaxation time of melanosomes^4^. The wavelength is selected in the visible to near-infrared range, which is absorbed by melanin and penetrates to the target^5^. The irradiated light experiences multiple scattering in the skin and its fluence is exponentially attenuated. At the target, melanosomes absorb the local fluence more efficiently than adjacent tissue components owing to higher absorption coefficients, resulting in selective disruption. However, the fluence in the skin is extremely high and normal blood vessels absorb multiple scattered light^6,7^, leading to complications, such as post-inflammatory hyperpigmentation, erythema, thermal burn, and scarring^8–10^. If it were feasible to disrupt deep target by setting the radiant exposure at a very low level, the damage to normal tissue caused by this treatment could be greatly reduced.

To disrupt a deep target with reduced damage to surrounding tissue, it is necessary to efficiently focus light into the target and provide a fluence greater than the threshold fluence for melanosome disruption^11^. A model of the melanosome-disruption threshold fluence (MDTF) has been proposed that considers the light-propagation efficiency in the skin^11^. This model can be used to calculate the lowest radiant exposure to deliver the threshold fluence to melanosomes in the skin and provide a numerical indicator to determine clinical irradiation conditions. However, the extent to which the radiation exposure required for laser treatment can be reduced is limited because the light propagation efficiency decays exponentially owing to multiple scattering by the skin. Thus, the model cannot describe the efficient focusing of light into a deep target.

Recently, wavefront-shaping techniques have been studied to compensate for optical aberrations and to control multiple scattering in biological tissues^12–14^. The use of these techniques enables the spatially selective focusing of light into a target, even at depths beyond the transport mean free path ( 1 mm)^15^. This breakthrough has led to improvements in the spatial resolution of optical imaging without invasive procedures^16^. In theory, if these techniques are applied to laser treatment, the radiant exposure at the skin surface will decrease in response to an increase in local fluence at the target. This means that by combining the MDTF model with a wavefront-shaping technique, an irradiation condition could be designed to increase the light-propagation efficiency to the target. Therefore, laser irradiation under such a designed irradiation condition has the potential to disrupt a deep target with a significant reduction in collateral tissue damage.

In this study, we demonstrate melanosome disruption with localized thermal damage through turbid media by irradiating a short-pulse laser with ultralow radiant exposure, which is lower than the lowest value determined using the MDTF model. The proposed method extends the MDTF model to increase the light-propagation efficiency to the target using a wavefront-shaping technique. With this approach, the light-propagation efficiency increases, resulting in a decrease in radiant exposure to disrupt melanosomes in the turbid medium. We found that melanosome disruption through the turbid medium occurred at a radiant exposure 25 times lower than conventional irradiation conditions, and that for ultralow radiant exposure, almost no thermal damage occurred in the skin tissue. Therefore, we expect the proposed method to contribute to improving the safety of short-pulsed laser treatment.

## Model of the melanosome-disruption threshold fluence

**Figure 1:**
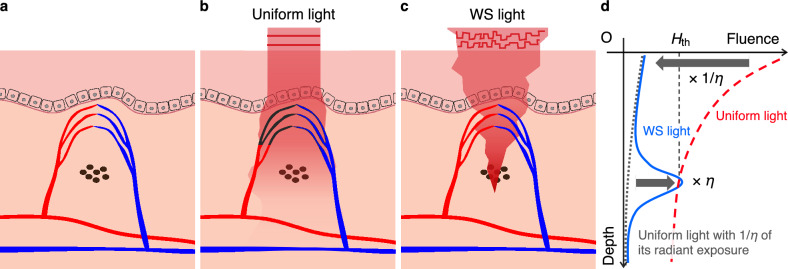
Concept of ultralow radiant exposure of a short-pulsed laser to disrupt melanosomes with localized thermal damage. (**a**) Before exposure. (**b**) In conventional laser treatments of dermal pigmented lesions, a short-pulsed laser with uniform phase distribution experiences multiple scattering by the skin (uniform light). The multiple scattered light is absorbed by the target as well as normal blood vessels, causing thermal damage to the adjacent tissue components. (**c**) In the proposed method, an irradiation condition is designed with the MDTF model and a wavefront-shaping technique to efficiently focus light to the target (WS light). The WS light can control multiple scattering by the skin and increase the light-propagation efficiency to the target, resulting in a decrease in radiant exposure. (**d**) The radiant exposure of WS light to disrupt melanosomes in the skin decreases with increasing light-propagation efficiency to the target.

Figure 1a illustrates skin tissue with pigmented lesions in the dermis. To provide a fluence greater than the disruption threshold fluence to the target, it is necessary to determine the radiant exposure considering the spatial distribution of melanosomes and the tissue’s optical properties. The radiant exposure, $F(\textbf{r})$ (**r**: position vector of target), required to deliver the threshold fluence to the target can be expressed by the following equation^11^:

1 $$\begin{aligned} F(\textbf{r}) \cdot \alpha (\varvec{\phi }, \textbf{r}) \ge H_{\text{th}}, \end{aligned}$$

where $\alpha$ is the light-propagation efficiency from the tissue surface to the target position **r** when a unit energy is irradiated (*i*.*e*., $\alpha$ is a unitless quantity), $\varvec{\phi }$ is the two-dimensional phase distribution of the incident laser, and $H_{\text{th}}$ is the threshold fluence for melanosome disruption. In conventional laser treatments, the phase of the incident laser is uniformly distributed as $\varvec{\phi }_u$, causing the light-propagation efficiency to exponentially decay with increasing depth. This increases the risk of thermal tissue damage because normal blood vessels above the lesions also absorb light (Fig. 1b). To decrease radiant exposure, it is necessary to increase the light-propagation efficiency to the target. Here, an improvement factor of the light-propagation efficiency, $\eta$, is introduced. Considering $\varvec{\phi }=\varvec{\phi }_\eta$ when the light-propagation efficiency is increased by a factor of $\eta$, the light-propagation efficiency can be expressed as $\alpha (\varvec{\phi }_\eta , \textbf{r})=\eta \alpha (\varvec{\phi }_u, \textbf{r})$. Substituting this into Eq. (1), the following equation is obtained:

2 $$\begin{aligned} \frac{F(\textbf{r})}{\eta } \cdot \alpha (\varvec{\phi }_\eta , \textbf{r}) \ge H_{\text{th}}. \end{aligned}$$

$F(\textbf{r})/\eta$ shows the radiant exposure when $\varvec{\phi }=\varvec{\phi }_\eta$. This allows for an $\eta$-fold reduction in radiant exposure compared with the case of $\varvec{\phi }=\varvec{\phi }_u$ (Fig. 1c,d). In this study, wavefront shaping was used to obtain the improvement factor. The radiant exposure of the wavefront-shaped (WS) light designed using the proposed method was compared with the lowest value of radiant exposure of uniform light in conventional laser treatment (*i.e.*, the radiant exposure determined by Eq. (1) for $\varvec{\phi }=\varvec{\phi }_u$).

## Results

### Ultralow radiant exposure of a short-pulsed laser to disrupt melanosomes through a turbid medium

**Figure 2:**
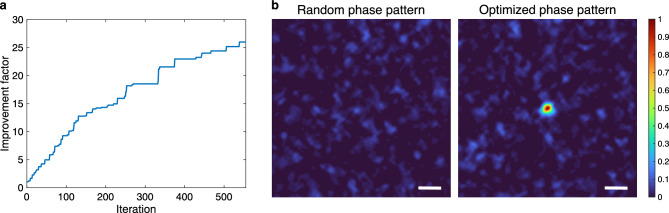
Experimental results showing an increase in the light-propagation efficiency to the target by wavefront shaping. (**a**) Improvement factor as a function of the number of iterations. (**b**) Light-intensity distributions at the target plane when a random phase pattern and the optimized phase pattern were displayed on the SLM. Scale bars: 10 μm.

**Figure 3:**
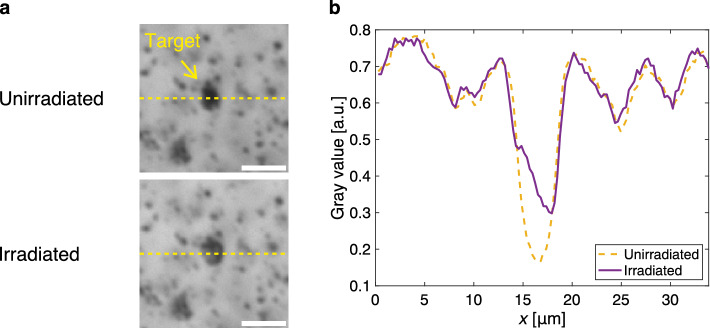
Comparison of a lesion phantom before and after picosecond laser irradiation. (**a**) Bright-field images of the unirradiated and irradiated melanosome aggregates. Scale bars: 10 μm. (**b**) Gray-value profiles on the dotted line in the bright-field images.

A picosecond laser was used in this experiment (see Methods section for details of the experimental setup). The wavelength was 532 nm because this study aimed to demonstrate the principle of melanosome disruption through a turbid medium at an ultralow radiant exposure using the extended MDTF model. A diffuser was used as the turbid medium. The pulsed light reflected from the spatial light modulator (SLM) was scattered by the diffuser. The incident beam size at the diffuser was 0.17 mm^2^. A lesion phantom prepared with melanosomes extracted from porcine eyes was placed at 2 mm behind the diffuser, corresponding to the depth of lesions^17^. When a uniform phase pattern was displayed on the SLM, the light-propagation efficiency to the target was 0.22. The disruption threshold of melanosomes using a 532-nm picosecond laser was 0.95 J/cm^2^^18^. Substituting these values into Eq. (1), the lowest value of radiant exposure of uniform light was determined to be 4.32 J/cm^2^. In the proposed method, the improvement factor was defined as the ratio of the fluence at the target to that at the target when a random phase pattern was displayed on the SLM. The optimization of the phase pattern was controlled with a genetic algorithm^19^. The light-intensity distribution at the target was used as the cost function and measured with a complementary metal-oxide-semiconductor (CMOS) camera. The optimization was terminated when the improvement fell below 5% over the last 100 iterations. The resulting improvement factor was 26 after 555 iterations (Fig. 2a). The spot size (full width at half maximum) was 3 μm (Fig. 2b). The radiant exposure of WS light was determined to be 0.17 J/cm^2^. To set the radiant exposure to this value, the optical density of the neutral-density filters and the angle of the half-wave plate were adjusted. Figure 3 shows the bright-field images of the lesion phantom before and after irradiation. The increase in gray value at the irradiated position shows the disruption of the melanosome aggregates.

### Validation of the irradiation condition designed using the extended MDTF model

**Figure 4:**
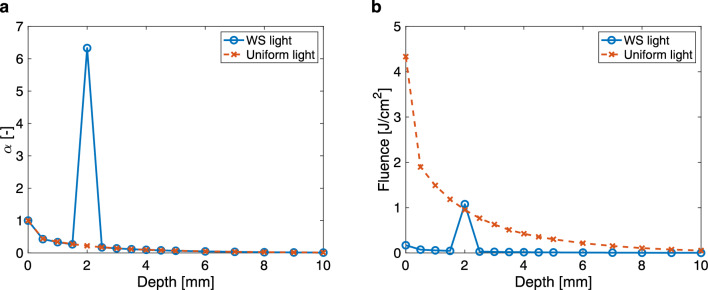
Validation of the increase in light-propagation efficiency and the local fluence at the target. (**a**) Depth profiles of the light-propagation efficiency for WS light and uniform light. (**b**) Depth profiles of the fluence for WS light and uniform light. The target was placed at 2 mm.

To verify whether the experimental irradiation condition matched that designed using the extended MDTF model, the light-propagation efficiency and the fluence at the target were obtained by displaying the optimized phase pattern on the SLM. The light-propagation efficiency at the target was 6.33, which was 29 times higher than that at the target when a uniform phase pattern was displayed on the SLM (Fig. 4a). From this, the local fluence at the target was estimated to be 1.08 J/cm^2^ (Fig. 4b). This value satisfied Eq. (2), therefore confirming the validity of the irradiation condition designed with the extended MDTF model.

### Evaluation of potential thermal damage to the tissue

**Figure 5:**
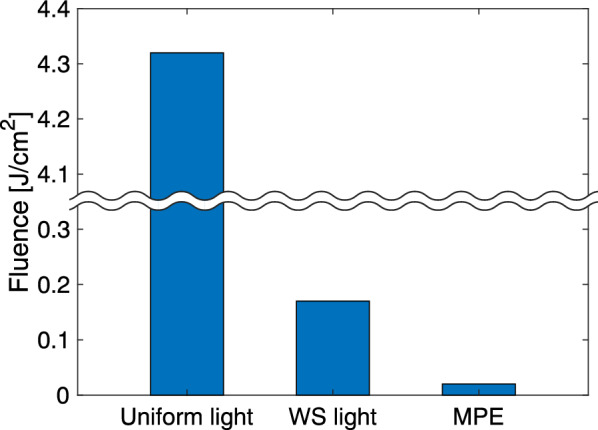
Comparison of the maximum fluence of WS light with that of uniform light and the MPE.

**Figure 6:**
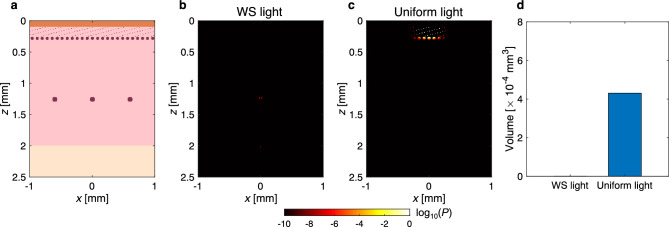
Comparison of the extent of thermal damage to the skin between WS light and uniform light. (**a**) Numerical tissue model consisting of the epidermis, dermis, subcutaneous fat, and blood vessels. (**b,c**) Thermal-damage distributions in the skin for WS light and uniform light. Thermal burn occurred at the blood vessels for uniform light. (**d**) Volumes of the thermally damaged tissue for WS light and uniform light. We note that the calculation of thermal damage for the WS light was performed assuming that the tissue was transparent and light was focused to the depth of the target to obtain the maximum thermal damage to normal tissue.

The potential tissue damage caused by laser irradiation was carefully investigated by comparing the fluence of WS light with that of uniform light and maximum permissible exposure (MPE), and numerically analyzing laser irradiation on skin tissue. First, the maximum fluences for the WS light and uniform light were compared (Fig. 5). The tissue region where the fluence peaked, excluding the lesion, was at the surface. The maximum fluence of WS light was 25 times smaller than that of uniform light. Furthermore, this value was 8.5 times larger than the MPE (0.02 J/cm^2^)^20^. Second, thermal burn of the skin tissue was analyzed through a numerical simulation of light propagation and thermal diffusion^21–23^. Optical absorption can be highly localized at structures such as blood vessels owing to high absorption coefficients. To consider this effect on the extent of thermal damage to the skin, a numerical model of human skin was assumed to have cylindrical structures of blood vessels at different depths (Fig. 6a)^23–25^. In the evaluation of the WS light, we considered a case in which the tissue was transparent and light was focused to the depth of the target to obtain the maximum thermal damage to normal tissue. Because the effect of scattering by the tissue is negligible in this case, the fluence becomes higher in the normal tissue above the target. In the evaluation of uniform light, a collimated beam was used rather than a focusing beam because the fluence in the normal tissue above the target becomes lower; therefore, the minimum thermal damage can be obtained. Figure 6b–d shows the thermal-damage distributions for the WS light and uniform light, and a comparison of the volumes of damaged tissue. Thermal burn of the blood vessels was observed for the uniform light, whereas there was almost no thermal damage to the normal tissue for the WS light. Therefore, we demonstrated that thermal damage could be localized within the target, without causing damage to the surrounding normal tissue.

## Discussion

This study demonstrated the disruption of melanosomes through scattering media at ultralow radiant exposure by improving the light-propagation efficiency to the target using a wavefront-shaping technique. Under ultralow radiant exposure, the radiant exposure of WS light was 25 times lower than that of uniform light, and almost no thermal damage was observed. The extent of the decrease in radiant exposure was almost identical to the extent of the increase in light-propagation efficiency at the target (*i*.*e*., the improvement factor). This implies that by estimating the light-propagation efficiency to the target in the initial state, it is possible to determine the improvement factor required to disrupt the target at a safe level of radiant exposure, such as the MPE. The initial light-propagation efficiency can be estimated using various computational methods, including Monte Carlo simulations^26^, diffusion approximations^27^, and beam-propagation methods^28^. The radiant exposure of WS light in this experiment was 8.5 times larger than the MPE. However, improvements of the proposed method may allow melanosome disruption at the MPE setting. To achieve this, the light-propagation efficiency to the target would need to be further increased by a factor of $\ge 8.5$. The theoretical value of the improvement factor is determined by $(N-1)\times \pi /4+1$, where *N* is the number of laser input modes^29^. The experimental value was lower than the theoretical value. This difference may be attributed to the fluctuation in the laser input modes, the uniformity in laser irradiation to the SLM, the accuracy of the optical alignment, and the parameter tuning of the optimization algorithm. Parameter-free optimization algorithms have been proposed, which showed an improvement factor 4.5 times higher than this experiment despite a lower number of input modes^30^. Combining the proposed method with such optimization algorithms may achieve selective disruption of melanosomes in a noninvasive manner at the MPE setting.

To efficiently disrupt melanosomes, it is necessary to optimize not only the radiant exposure but also the pulse width and wavelength. Picosecond-to-nanosecond pulse widths are commonly used because they satisfy the thermal-confinement condition of melanosomes. At a high power density, melanosomes absorb light in a nonlinear manner and the absorption efficiency varies with pulse width. Owing to this effect, melanosomes efficiently absorb light in the picosecond regime^31^; therefore, a picosecond laser was used in this experiment. A wavelength of 532 nm is commonly used for treating epidermal pigmented lesions because of its low penetrability in skin tissue^32^. However, light at 532 nm requires a high fluence for treating dermal pigmented lesions, which may lead to thermal damage to blood vessels owing to optical absorption by hemoglobin. Typically, near-infrared wavelengths with relatively high optical absorption by melanin and high tissue penetrability are used instead^33^. Wavelength optimization may lead to disruption of deeper melanosomes with ultralow radiant exposure.

It is important to evaluate potential thermal damage to structures such as blood vessels because optical absorption is highly localized at these structures. Skin models commonly used in diffuse reflectance spectroscopy studies are constructed with a homogeneous structure based on the volume fraction of hemoglobin^34,35^. These models may underestimate thermal damage to blood vessels and overestimate thermal damage to surrounding tissue in laser treatment simulations. Therefore, a skin model with vascular structures at different depths was constructed in our numerical simulation to evaluate the effect of vascular structures on the extent of thermal damage. Although our simulation assumed vascular structures to be cylindrical, real blood vessels in the skin have a more complex branching distribution. If this complex branching distribution is considered in the skin model, it may increase the volume of damaged tissue compared to the current skin model. To address this, a previous study constructed a skin model with vascular structures measured using optical coherence tomography^24^. The use of such a skin model could improve the accuracy of the analysis of local heating effects.

In this study, the phase pattern on the SLM was calculated using the light-intensity distribution at the target plane. Because this signal does not contain information about the target, the applicability of this method to lesions within tissue is limited. To overcome this limitation, indirect sensing of the light intensity at the target is required using guide stars, such as photoacoustic, fluorescence, or ultrasound encoding, depending on the target disease. Various wavefront-shaping techniques based on these guide stars can be applied to increase the light-propagation efficiency to the target, including iterative wavefront optimization and optical phase conjugation^36^. This application will improve the performance of the proposed method. Photoacoustic-guided wavefront shaping is preferable when the target is a chromophore, such as pigmented lesions, melanoma, or abnormal blood vessels^37^. Fluorescence-guided wavefront shaping is effective for fluorophores, such as tumor cells with accumulated photosensitizers, improving the spatial selectivity of photodynamic therapy^38^. In cases where the target has less optical absorption, ultrasound encoding can create virtual guide stars, enabling the focusing of light into the target^39^.

Other challenges occur when the proposed method is applied to the skin tissue. One particular challenge is the transmission configuration of the experimental setup. Since detecting the target from the other side of the incident laser is not feasible owing to the thickness of the tissue, a reflective configuration needs to be constructed. Another challenge is the slow speed of optimization. In this experiment, it took  2.5 h to determine the irradiation condition, with most of the time spent to optimizing the phase pattern on the SLM. Because of this long optimization time, a stable diffuser was used to demonstrate the principle of melanosome disruption with localized thermal damage at ultralow radiant exposure. However, scattering in vivo fluctuates owing to physiological motion, such as blood flow and respiration. All steps must be completed within the speckle decorrelation time (0.1–10 ms)^40^. Future studies should use fast SLMs to accelerate the optimization. We plan to continue this work to overcome these limitations by performing experiments with a reflective configuration using a fast SLM to make the proposed method applicable to treating thick tissue.

In conclusion, this study demonstrated the principle of melanosome disruption with localized thermal damage through a turbid medium at ultralow radiant exposure of a short-pulsed laser. In the proposed method, the radiant exposure was 25 times lower than the minimal value in conventional laser treatments. The experimental and numerical results demonstrated the potential of selective disruption of melanosomes in a noninvasive way and will contribute to the improvement of the safety of short-pulsed laser treatment. Although there are limitations in the application to the target inside a turbid medium, these could be overcome by using photoacoustic-guided wavefront shaping.

## Methods

### Sample preparation

A lesion phantom was prepared using porcine eye melanosomes as an alternative sample to human skin melanosomes^11,18^. Porcine eye melanosomes were extracted using a method similar to that reported elsewhere^41^. Porcine eyes were dissected, and the lens and vitreous were removed. The retina was detached and then suspended in distilled water. The melanosomes in the suspension were extracted using a toothbrush. The suspension was purified with a 2.7-μm pore filter. To remove cell debris and blood, the purified suspension was centrifuged at 3000 rpm for 20 min. After that, the supernatant was removed and the melanosomes were resuspended in distilled water. Gelatin (10% w/v, G2500, Sigma-Aldrich) was added to the suspension and heated in a water bath at 37 °C for 30 min. The samples were adjusted to a thickness of 0.1 mm using two slide glasses and spacers, and stored at 4 °C for 12 h to solidify.

### Experimental setup

The experimental setup is schematically shown in Supplementary Fig. S1. A 532-nm microchip laser (CLM05-01-050-T, Optoquest) was used, which produced 0.6-ns pulses (pulse energy $\ge$ 1 mJ) at a pulse repetition rate of 20–50 Hz. The laser energy was adjusted using a half-wave plate, a polarizing beam splitter, and neutral-density filters. The light reflected from the beam splitter was directed onto another half-wave plate, and was then reflected from a liquid crystal on silicon SLM (E19$\times$12–500–1200–HDMI–TT, Meadowlark Optics). This half-wave plate adjusted the angle of polarization incident on the SLM. The resolution of the SLM was 8 μm and the number of pixels was $1920 \times 1200$. In the experiment, the SLM was divided evenly into blocks of $192 \times 120$, and the phase was shifted between 0 and 2$\pi$. The reflected light was focused onto a diffuser (DG10-120, Thorlabs) by a condenser lens ($f=30$ mm). The light-propagation efficiency of the diffuser was equivalent to that of human skin tissue (see Supplementary Fig. S2). The light-intensity distribution on the target plane, 2 mm from the diffuser, was measured as the cost function. The imaging system consisted of an objective lens ($\times 10$, $\text{NA}=0.25$), a tube lens, and a CMOS camera (resolution: 2.74 μm/pixel, number of pixels: $2448 \times 2048$). The laser and camera shutters were synchronized using a digital delay generator (DG535, Stanford Research Systems). The phase pattern on the SLM was optimized with a genetic algorithm^19^. The improvement factor was defined as the ratio of the fluence at the target to that at the target for a random phase pattern on the SLM. The light intensity was almost identical for the random and uniform phase patterns on the SLM, suggesting that the wavefront of the incident laser was randomized by the diffuser. In addition, the light-propagation efficiency of the diffuser was almost the same for the random and uniform phase patterns on the SLM.

### Measurement of light-propagation efficiency

The light-propagation efficiency was obtained by normalizing the fluence with varying depth to the fluence at the surface of the diffuser. The imaging system was moved using a manual stage with an accuracy of 0.01 mm. The light-propagation efficiency was measured at intervals of 0.5 mm to a depth of 5 mm from the diffuser and at intervals of 1.0 mm beyond 5 mm. A uniform phase pattern was displayed on the SLM in the case of uniform light, whereas the optimized phase pattern was displayed in the case of WS light.

### Numerical simulation of light propagation and thermal diffusion

A three-dimensional numerical tissue model, consisting of the epidermis, dermis, subcutaneous fat, and blood vessels, was constructed using $400 \times 400 \times 500$ voxels^23^. The voxel was a cube with 5-μm sides. The depth direction was set along the *z* axis, with the tissue surface at *z* = 0 mm. The plane vertical to the *z* axis was defined as the *xy* plane, and the center on the *xy* plane was set as (*x*, *y*) = (0 mm, 0 mm). The thicknesses of the epidermis and the dermis were set at 0.1 and 1.9 mm, respectively. The structural parameters of blood vessels are shown in Supplementary Table S1. Optical and thermal properties of the human skin tissue were input to the numerical model, as shown in Supplementary Table S2. A Monte Carlo simulation was used to calculate the energy-deposition distribution in the tissue^26^. To evaluate the maximum thermal damage to normal tissue by the WS light, we considered a case in which the tissue was transparent and light was focused to the depth of the target (2 mm). Because the effect of scattering by the tissue is negligible in this case, the fluence was highest in the normal tissue above the target. The reduced scattering coefficient and anisotropy factor of the skin were changed to 0.1 mm^−1^ and 1.0, respectively. The spot size at the target was 5 μm. The radiant exposure was set as 0.17 J/cm^2^. In the case of uniform light, the minimum thermal damage to normal tissue was evaluated. A collimated beam was used instead of a focusing beam because the fluence in the normal tissue above the target was lower for a collimated beam. The radiant exposure was set as 4.32 J/cm^2^. In both cases, the beam profile was a Gaussian distribution and the incident beam size was 0.17 mm^2^, similar to the experiment. The number of photon packets was $10^9$. The temperature rise in the tissue was calculated using the thermal-diffusion equation:

3 $$\begin{aligned} \rho c \frac{\partial T(\textbf{r},t)}{\partial t} = k \nabla ^2 T(\textbf{r},t) + S(\textbf{r},t), \end{aligned}$$

where $\rho$ is the density, *c* is the specific heat capacity, *k* is the thermal conductivity and *S* is the energy deposition. This equation was solved using the finite-difference method. At the tissue surface, the convective boundary condition was considered:

4 $$\begin{aligned} k \frac{\partial T(z=0,t)}{\partial z} = h (T_\text{air}-T(z=0,t) ), \end{aligned}$$

where *h* is the thermal-transfer coefficient (0.001 W/($\hbox {cm}^2\cdot$K))^23^ and $T_\text{air}$ is the temperature of the air (22 °C). The adiabatic condition was applied to the other boundaries. The initial temperature for the tissue was set at 37 °C. The extent of thermal damage is dependent not only on the temperature but also on the tissue’s exposure time at a given temperature^31^. The Arrhenius integral was therefore used to evaluate thermal damage:

5 $$\begin{aligned} \Omega (\textbf{r},t) = \ln \left\{ \frac{C(\textbf{r},0)}{C(\textbf{r},t)} \right\} = A \displaystyle \int _0^t \exp {\left\{ \frac{E_\text{{a}}}{R T(\textbf{r},t)} \right\} } \text{{d}}t, \end{aligned}$$

where $C(\textbf{r},t)$ is the remaining concentration of undamaged tissue, *A* is the frequency factor, $E_\text{a}$ is the activation energy, and *R* is the gas constant. The relative concentration (*i*.*e*., probability) of damaged tissue, *P*, was calculated using the following equation:

6 $$\begin{aligned} P = 1-\exp \{-\Omega (\textbf{r},t)\}. \end{aligned}$$

In this experiment, $P=63.2\%$ (*i.e.*, $\Omega = 1$) was applied as the threshold for thermal burn, including cell necrosis and protein coagulation^42^. The volume of the thermally damaged tissue was compared between the WS light and uniform light.

### Supplementary Information

Supplementary Information.

## Data Availability

The datasets used and/or analyzed during the current study are available from the corresponding author on reasonable request.
