# Supplementary material for: Ultralow radiant exposure of a short-pulsed laser to disrupt melanosomes with localized thermal damage through a turbid medium
Source: Sci Rep. 2024 Aug 29;14:20112. doi: 10.1038/s41598-024-70807-7 (PMC11362287; doi:10.1038/s41598-024-70807-7)
Supplement: Supplementary file 1 — Supplementary Information. [file 41598_2024_70807_MOESM1_ESM.docx]

**Ultralow radiant exposure of a short-pulsed laser to disrupt melanosomes with localized thermal damage through a turbid medium**

Yu Shimojo^1,2,3^, Takahiro Nishimura^2^, Daisuke Tsuruta^1^, Toshiyuki Ozawa^1^

^1^Graduate School of Medicine, Osaka Metropolitan University, Asahimachi 1-4-3, Abeno-ku, Osaka, 545-8585, Japan

^2^Graduate School of Engineering, Osaka University, Yamadaoka 2-1, Suita, Osaka, 565-0871, Japan

^3^Research Fellow of Japan Society for the Promotion of Science, Kojimachi 5-3-1, Chiyoda-ku, Tokyo, 102-0083, Japan

**Supplementary Information**

**Experimental setup**

**
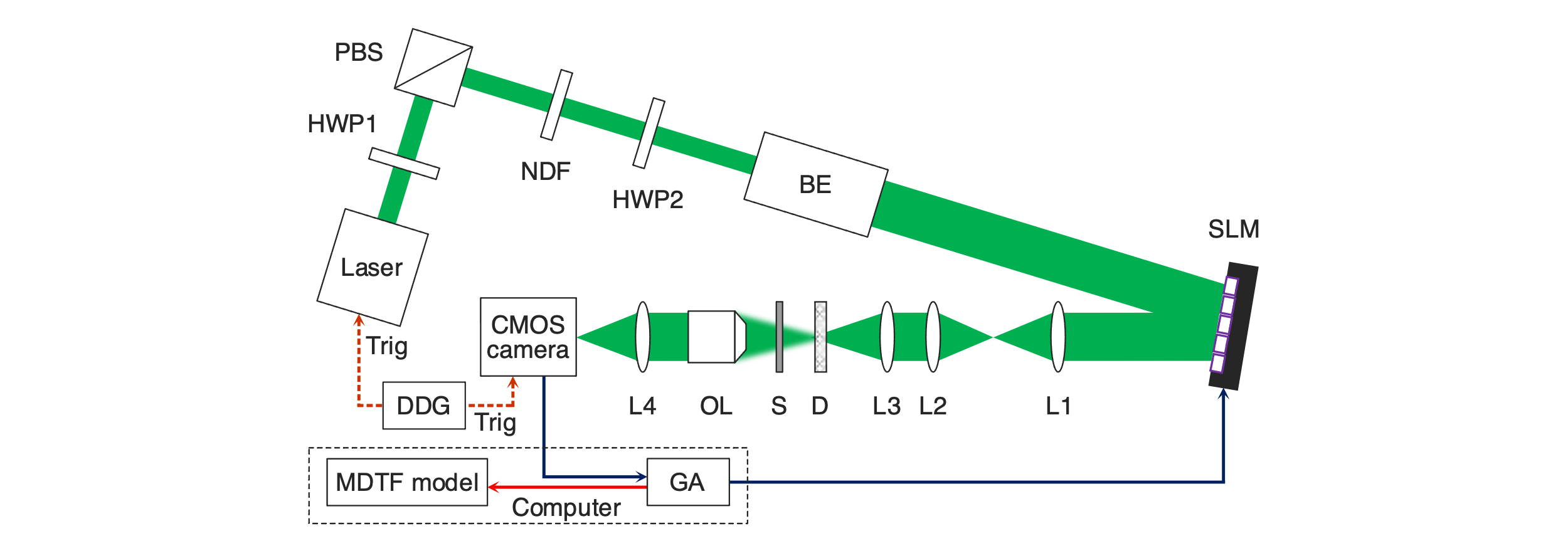
**

**Figure S1**. Schematic illustration of the experimental setup. BE, beam expander; D, diffuser; DDG, digital delay generator; GA, genetic algorithm; HWP1 and HWP2, half-wave plates; L1–L4, lenses; MDTF model, melanosome-disruption threshold fluence model; NDF, neutral-density filters; OL, objective lens; PBS, polarization beam splitter; S, sample; SLM, spatial light modulator; Trig, trigger signal.

**Calculation of light-propagation efficiency in the skin**

The light-propagation efficiency in the skin was calculated using the following equation:

| $\alpha_{s\mathrm{kin}}\left( z \right)=\exp\left( -\sqrt{3\mu_{a}\left( \mu_{a}+\mu_{s}^{'} \right)} z \right),$ | (S1) |
| --- | --- |

where *μ*_a_ is the absorption coefficient of human skin and *μ*_s_′ is the reduced scattering coefficient of human skin (Supplementary Table S2). Figure S1 shows the light-propagation efficiencies of the diffuser and the skin. The light-propagation efficiency to the depth of the target (2 mm) was similar for the diffuser and the skin.


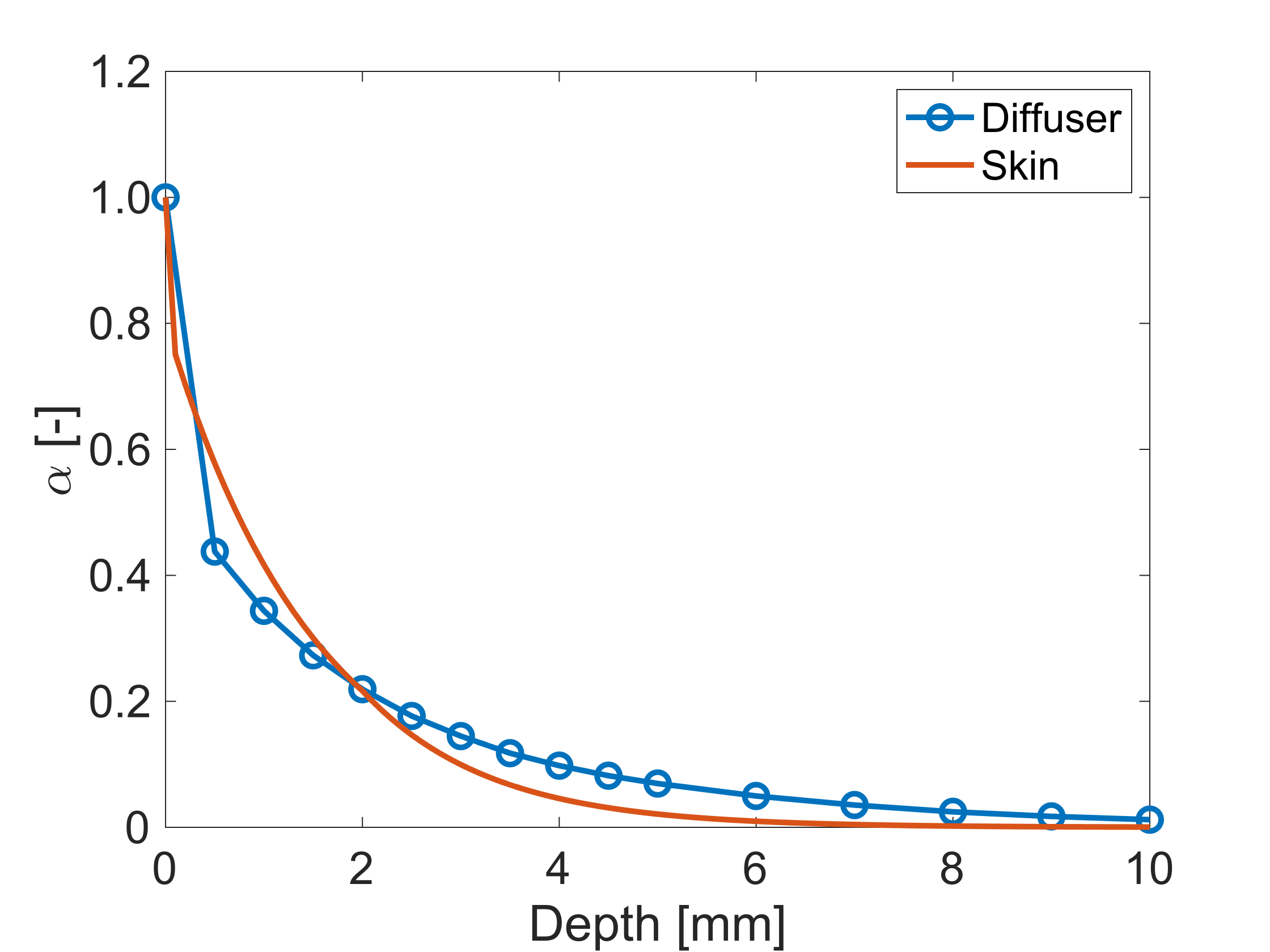


**Figure S2.** Light-propagation efficiency of the diffuser (measured) and the skin (calculated).

**Numerical simulation of light propagation and thermal diffusion**

**Table S1.** Structural parameters used for blood vessels^1^.

|  | Center depth (μm] | Volume fraction (%) | Diameter (μm) |
| --- | --- | --- | --- |
| Capillary plexus | 175 | 4 | 10 |
| Upper dermis blood vessels | 290 | 30 | 50 |
| Deep dermis blood vessels | 1260 | 10 | 80 |

**Table S2.** Optical and thermal properties of the numerical tissue model^2–8^.

| Parameter | | Epidermis | Dermis | Fat | Blood |
| --- | --- | --- | --- | --- | --- |
| Absorption coefficient *μ*_a_ (mm^−1^) | | 0.77 | 0.05 | 0.14 | 23.43 |
| Reduced scattering coefficient *μ*_s_′ (mm^−1^) | | 2.8 | 2.8 | 1.3 | 21.1 |
| Anisotropy factor *g* (-) | | 0.9 | | | |
| Density *ρ* (g/cm^3^) | | 1.19 | 1.12 | 0.97 | 1.00 |
| Specific heat capacity *c* (J/(g·K)) | | 3.60 | 3.22 | 2.30 | 3.84 |
| Thermal conductivity *k* (×10^−3^ W/(cm·K)) | | 2.09 | 3.00 | 2.05 | 4.92 |
| Frequency factor *A* (1/s) | *T* ≤ 53 °C | 8.82×10^94^ | | | |
|  | *T* > 53 °C | 1.297×10^31^ | | | |
| Activation energy *E*_a_ (J/mol) | *T* ≤ 53 °C | 6.028×10^5^ | | | |
|  | *T* > 53 °C | 2.04×10^5^ | | | |
| Gas constant *R* (J/(mol·K)) | | 8.31 | | | |

**References**

1. Meglinski, I. V. & Matcher, S. J. Computer simulation of the skin reflectance spectra. *Comput. Methods Programs. Biomed*. **70**, 179–186 (2003).
2. Shimojo, Y., Nishimura, T., Hazama, H., Ito, N. & Awazu, K. Picosecond laser-induced photothermal skin damage evaluation by computational clinical trial. *Laser Ther.* **29**, 61–72 (2020).
3. Jacques, S. L. Optical properties of biological tissues: a review. *Phys. Med. Biol.* **58**, R37–R61 (2013).
4. Bashkatov, A. N., Genina, E. A. & Tuchin, V. V. Optical properties of skin, subcutaneous, and muscle tissues: a review. *J. Innov. Opt. Health Sci*. **4**, 9–38 (2011).
5. Simpson, C. R., Kohl, M., Essenpreis, M. & Cope, M. Near infrared optical properties of *ex-vivo* human skin and subcutaneous tissues measured using the Monte Carlo inversion technique. *Phys. Med. Biol.* **43**, 2465–2478 (1998).
6. Tuchin, V. V. *Tissue Optics: Light Scattering Methods and Instruments for Medical Diagnosis* (SPIE, 2007), 2 edn.
7. Jacques S. L. Optical Absorption of Melanin. [Accessed 2024 Apr 30]. Available from: https://omlc.org/news/jan98/skinoptics.html
8. Pearce, J. A. Relationship between Arrhenius models of thermal damage and the CEM 43 thermal dose. *Proc. SPIE*. **7181**, 718104 (2009).
